# Bis[μ-3-(2-hydroxy­ethyl)-2-methyl-4-oxo-4*H*-pyrido[1,2-*a*]pyrimidin-9-olato-κ^3^ *N*,*O*:*O*]bis[aquachloridocopper(II)]

**DOI:** 10.1107/S1600536808028687

**Published:** 2008-09-13

**Authors:** Ying Deng, Zhong-Shu Li, Bai-Wang Sun

**Affiliations:** a Ordered Matter Science Research Center, College of Chemistry and Chemical Engineering, Southeast University, Nanjing 210096, People’s Republic of China

## Abstract

In the dinuclear centrosymmetric copper(II) title compound, [Cu_2_(C_11_H_11_N_2_O_2_)_2_Cl_2_(H_2_O)_2_], each Cu^II^ ion has a slightly distorted trigonal-bipyramidal geometry and is coordinated by one N and one O atom from one 3-(2-hydroxy­ethyl)-2-methyl-4-oxopyrido[1,2-*a*]pyrimidin-9-olate ligand, another O atom from the second ligand, one water mol­ecule and one Cl atom. The crystal structure involves inter­molecular C—H⋯Cl, O—H⋯Cl and O—H⋯O hydrogen bonds

## Related literature

For related literature, see: Bayot *et al.* (2006 ▶); Chen *et al.* (2007 ▶); Sun *et al.* (2008 ▶); Wu *et al.* (2006 ▶).
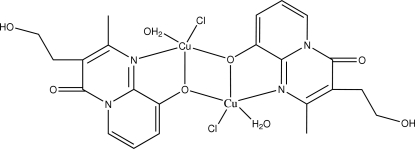


## Experimental

### 

#### Crystal data

[Cu_2_(C_11_H_11_N_2_O_2_)_2_Cl_2_(H_2_O)_2_]
*M* *_r_* = 672.45
Monoclinic, 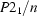

*a* = 9.391 (3) Å
*b* = 11.322 (3) Å
*c* = 11.905 (4) Å
β = 102.414 (18)°
*V* = 1236.2 (6) Å^3^
*Z* = 2
Mo *K*α radiation
μ = 1.99 mm^−1^
*T* = 293 (2) K
0.25 × 0.12 × 0.08 mm

#### Data collection

Rigaku SCXmini diffractometer
Absorption correction: multi-scan (*CrystalClear*; Rigaku, 2005 ▶) *T*_min_ = 0.890, *T*_max_ = 1.000 (expected range = 0.759–0.853)
12472 measured reflections
2826 independent reflections
2249 reflections with *I* > 2σ(*I*)
*R*_int_ = 0.056

#### Refinement

*R*[*F* ^2^ > 2σ(*F* ^2^)] = 0.045
*wR*(*F* ^2^) = 0.115
*S* = 1.01
2826 reflections
176 parameters
H atoms treated by a mixture of independent and constrained refinement
Δρ_max_ = 0.49 e Å^−3^
Δρ_min_ = −0.47 e Å^−3^

### 

Data collection: *CrystalClear* (Rigaku, 2005 ▶); cell refinement: *CrystalClear*; data reduction: *CrystalClear*; program(s) used to solve structure: *SHELXS97* (Sheldrick, 2008 ▶); program(s) used to refine structure: *SHELXL97* (Sheldrick, 2008 ▶); molecular graphics: *SHELXTL* (Sheldrick, 2008 ▶); software used to prepare material for publication: *SHELXL97*.

## Supplementary Material

Crystal structure: contains datablocks I, global. DOI: 10.1107/S1600536808028687/sg2256sup1.cif

Structure factors: contains datablocks I. DOI: 10.1107/S1600536808028687/sg2256Isup2.hkl

Additional supplementary materials:  crystallographic information; 3D view; checkCIF report

## Figures and Tables

**Table 1:** Hydrogen-bond geometry (Å, °)

*D*—H⋯*A*	*D*—H	H⋯*A*	*D*⋯*A*	*D*—H⋯*A*
O5—H5*C*⋯O2^i^	0.82	2.13	2.742 (4)	131
O5—H5*D*⋯O1^ii^	0.96	2.11	2.788 (4)	127
O2—H2*A*⋯Cl1^iii^	0.75 (6)	2.37 (6)	3.078 (4)	158 (6)
C9—H9*A*⋯Cl1	0.96	2.49	3.275 (4)	139
C3—H3*A*⋯Cl1^iv^	0.93	2.72	3.387 (4)	130

Symmetry codes: (i) 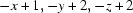; (ii) 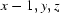; (iii) 
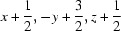
; (iv) 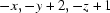.

**Fig. 1.**
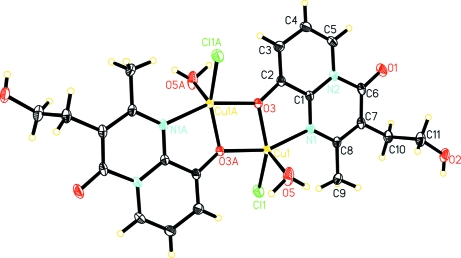
The molecular structure of the title compound with the atom-numbering scheme and all hydrogen atoms. Displacement ellipsoids are drawn at the 30% probability level.

**Fig. 2.**
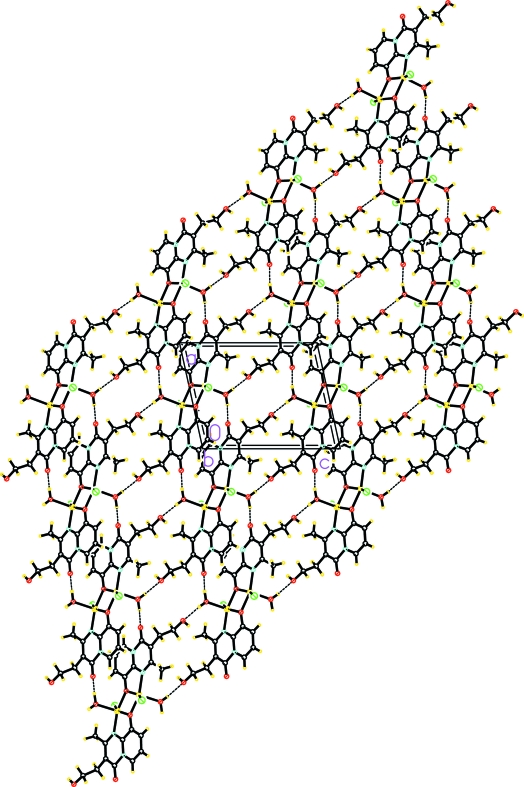
Two-dimensional net framework of the title compound viewed along b axis. Hydrogen bonds are shown as dashed lines. Displacement ellipsoids are drawn at the 30% probability level.

**Fig. 3.**
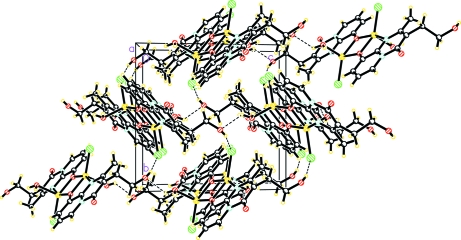
Three-dimensional net framework of the title compound viewed along a axis. Hydrogen bonds are shown as dashed lines. Displacement ellipsoids are drawn at the 30% probability level.

## supplementary crystallographic information

### Comment

In the past decade, much attention has been paid to the design and synthesis of self-assembling systems with organic ligands containing N and O donors (Bayot *et al.*, 2006; Chen *et al.*, 2007). Quinolin-8-ol is one such ligand and several crystal structures of complexes containing it have been reported (Wu *et al.*, 2006). Our group has recently reported a new manganese compound with this 3-(2-hydroxyethyl)-2-methyl-4-oxopyrido[1,2-*a*]pyrimidin-9-olate ligands (Sun *et al.*, 2008). We report here the synthesis and crystal structure of the title complex, (I) (Fig. 1). In (I), each Cu(II) ion has a slightly distorted trigonal–bipyramidal geometry and is coordinated by one N atoms and one O atom from one 3-(2-hydroxyethyl)-2-methyl-4-oxopyrido[1,2-*a*]pyrimidin-9-olate ligand, the another O atom of the second 3-(2-hydroxyethyl)-2-methyl-4-oxopyrido[1,2-*a*]pyrimidin-9-olate ligand, together with one water molecule and one Cl atom (Fig. 1). The bond lengths and angles are shown in Table 1. In the crystal structure, the intermolecular O—H···O hydrogen bonds connect the molecules of (I) into a two-dimensional layer along the [010] axis, Fig. 2. Two neighboring net framework layers are interconnected through intermolecular C—H···Cl, O—H···Cl hydrogen bonds forming a three-dimensionnal framework along the [100] axis, Fig. 3, Table 2.

### Experimental

All chemicals used (reagent grade) were commercially available. a aqueous solution (5 ml) of CuCl_2_ (28 mg, 0.1 mmol) was added with constant stirring to a ethanol solution (10 ml) containing 3-(2-hydroxyethyl)-2-methyl-9-hydroxylpyrido[1,2-*a*] pyrimidin-4-one (22 mg, 0.1 mmol) then filtered off. After a few days, colorless well shaped single crystals in the form of prisms deposited in the other liquid. They were separated off, washed with cold ethanol and dried in air at room temperature.

### Refinement

H atoms bound to carbon were included in calculated positions and treated in the subsequent refinement as riding atoms, with C—H = 0.94 Å and *U*_iso_(H) = 1.2*U*_eq_(C). The H of hydroxyl and water were refined independently with isotropic displacement parameters.

### Crystal data

**Table:**

[Cu_2_(C_11_H_11_N_2_O_2_)_2_Cl_2_(H_2_O)_2_]	*F*(000) = 684
*M**_r_* = 672.45	*D*_x_ = 1.807 Mg m^−^^3^
Monoclinic, *P*2_1_/*n*	Mo *K*α radiation, λ = 0.71073 Å
Hall symbol: -P 2yn	Cell parameters from 2976 reflections
*a* = 9.391 (3) Å	θ = 2.5–27.5°
*b* = 11.322 (3) Å	µ = 1.99 mm^−^^1^
*c* = 11.905 (4) Å	*T* = 293 K
β = 102.414 (18)°	Prism, colorless
*V* = 1236.2 (6) Å^3^	0.25 × 0.12 × 0.08 mm
*Z* = 2	

### Data collection

**Table:**

Rigaku SCXmini diffractometer	2826 independent reflections
Radiation source: fine-focus sealed tube	2249 reflections with *I* > 2σ(*I*)
graphite	*R*_int_ = 0.056
Detector resolution: 8.192 pixels mm^-1^	θ_max_ = 27.5°, θ_min_ = 2.5°
ω scans	*h* = −12→12
Absorption correction: multi-scan (CrystalClear; Rigaku, 2005)	*k* = −14→14
*T*_min_ = 0.890, *T*_max_ = 1.000	*l* = −15→15
12472 measured reflections	

### Refinement

**Table:**

Refinement on *F*^2^	Primary atom site location: structure-invariant direct methods
Least-squares matrix: full	Secondary atom site location: difference Fourier map
*R*[*F*^2^ > 2σ(*F*^2^)] = 0.045	Hydrogen site location: inferred from neighbouring sites
*wR*(*F*^2^) = 0.115	H atoms treated by a mixture of independent and constrained refinement
*S* = 1.02	*w* = 1/[σ^2^(*F*_o_^2^) + (0.059*P*)^2^ + 0.8671*P*] where *P* = (*F*_o_^2^ + 2*F*_c_^2^)/3
2826 reflections	(Δ/σ)_max_ = 0.001
176 parameters	Δρ_max_ = 0.49 e Å^−^^3^
0 restraints	Δρ_min_ = −0.47 e Å^−^^3^

### Special details

**Table:**

Geometry. All e.s.d.'s (except the e.s.d. in the dihedral angle between two l.s. planes) are estimated using the full covariance matrix. The cell e.s.d.'s are taken into account individually in the estimation of e.s.d.'s in distances, angles and torsion angles; correlations between e.s.d.'s in cell parameters are only used when they are defined by crystal symmetry. An approximate (isotropic) treatment of cell e.s.d.'s is used for estimating e.s.d.'s involving l.s. planes.
Refinement. Refinement of *F*^2^ against ALL reflections. The weighted *R*-factor *wR* and goodness of fit *S* are based on *F*^2^, conventional *R*-factors *R* are based on *F*, with *F* set to zero for negative *F*^2^. The threshold expression of *F*^2^ > σ(*F*^2^) is used only for calculating *R*-factors(gt) *etc*. and is not relevant to the choice of reflections for refinement. *R*-factors based on *F*^2^ are statistically about twice as large as those based on *F*, and *R*- factors based on ALL data will be even larger.

### Fractional atomic coordinates and isotropic or equivalent isotropic displacement parameters (Å^2^)

**Table:**

	*x*	*y*	*z*	*U*_iso_*/*U*_eq_	
Cu1	0.09275 (4)	0.92674 (4)	0.60256 (3)	0.02472 (15)	
C1	0.3469 (3)	1.0520 (3)	0.5971 (3)	0.0230 (7)	
C2	0.2373 (4)	1.1028 (3)	0.5076 (3)	0.0241 (7)	
C3	0.2761 (4)	1.1877 (3)	0.4388 (3)	0.0315 (8)	
H3A	0.2069	1.2198	0.3788	0.038*	
C4	0.4213 (4)	1.2264 (3)	0.4592 (3)	0.0335 (8)	
H4A	0.4474	1.2855	0.4133	0.040*	
C5	0.5234 (4)	1.1795 (3)	0.5441 (3)	0.0309 (8)	
H5A	0.6190	1.2067	0.5566	0.037*	
C6	0.6009 (4)	1.0432 (3)	0.7013 (3)	0.0294 (8)	
C7	0.5562 (4)	0.9521 (3)	0.7664 (3)	0.0284 (8)	
C8	0.4116 (4)	0.9186 (3)	0.7479 (3)	0.0259 (7)	
C9	0.3610 (4)	0.8284 (3)	0.8226 (3)	0.0342 (9)	
H9A	0.2576	0.8176	0.7978	0.051*	
H9B	0.3833	0.8550	0.9010	0.051*	
H9C	0.4097	0.7548	0.8169	0.051*	
C10	0.6746 (4)	0.8972 (3)	0.8572 (3)	0.0319 (8)	
H10A	0.6415	0.8209	0.8786	0.038*	
H10B	0.7601	0.8841	0.8254	0.038*	
C11	0.7161 (5)	0.9729 (4)	0.9631 (3)	0.0421 (10)	
H11A	0.7600	1.0453	0.9432	0.051*	
H11B	0.6285	0.9939	0.9893	0.051*	
O1	0.7242 (3)	1.0832 (2)	0.7112 (3)	0.0412 (7)	
O2	0.8149 (3)	0.9165 (3)	1.0542 (3)	0.0441 (8)	
O3	0.1054 (2)	1.0586 (2)	0.4999 (2)	0.0313 (6)	
O5	0.0115 (3)	1.0044 (3)	0.7446 (2)	0.0542 (9)	
H5C	0.0633	0.9835	0.8059	0.081*	
H5D	−0.0866	0.9780	0.7408	0.081*	
N1	0.3065 (3)	0.9674 (2)	0.6621 (2)	0.0244 (6)	
N2	0.4871 (3)	1.0915 (2)	0.6127 (2)	0.0253 (6)	
Cl1	0.07956 (10)	0.73242 (8)	0.63230 (9)	0.0408 (3)	
H2A	0.775 (6)	0.878 (5)	1.088 (5)	0.07 (2)*	

### Atomic displacement parameters (Å^2^)

**Table:**

	*U*^11^	*U*^22^	*U*^33^	*U*^12^	*U*^13^	*U*^23^
Cu1	0.0176 (2)	0.0284 (2)	0.0255 (2)	−0.00189 (17)	−0.00112 (16)	0.00366 (16)
C1	0.0188 (16)	0.0241 (16)	0.0247 (17)	−0.0006 (13)	0.0013 (13)	−0.0017 (13)
C2	0.0168 (15)	0.0286 (17)	0.0260 (17)	0.0011 (13)	0.0022 (13)	−0.0013 (13)
C3	0.0283 (18)	0.0332 (19)	0.032 (2)	0.0011 (16)	0.0041 (15)	0.0050 (15)
C4	0.0291 (18)	0.032 (2)	0.040 (2)	−0.0036 (16)	0.0079 (16)	0.0057 (16)
C5	0.0222 (17)	0.0316 (19)	0.039 (2)	−0.0077 (15)	0.0074 (15)	−0.0011 (15)
C6	0.0162 (16)	0.0350 (19)	0.0337 (19)	0.0002 (14)	−0.0021 (14)	−0.0057 (15)
C7	0.0195 (16)	0.0326 (19)	0.0299 (18)	0.0024 (14)	−0.0019 (14)	−0.0050 (14)
C8	0.0214 (16)	0.0276 (17)	0.0253 (17)	0.0036 (14)	−0.0025 (13)	−0.0005 (13)
C9	0.0294 (19)	0.041 (2)	0.0278 (19)	0.0015 (16)	−0.0038 (15)	0.0064 (15)
C10	0.0212 (17)	0.035 (2)	0.035 (2)	0.0064 (15)	−0.0023 (15)	−0.0025 (15)
C11	0.037 (2)	0.041 (2)	0.040 (2)	0.0059 (18)	−0.0092 (17)	−0.0046 (18)
O1	0.0170 (12)	0.0495 (17)	0.0534 (18)	−0.0039 (12)	−0.0007 (12)	0.0014 (13)
O2	0.0312 (16)	0.061 (2)	0.0326 (16)	0.0038 (15)	−0.0087 (13)	0.0010 (14)
O3	0.0160 (11)	0.0413 (15)	0.0321 (14)	−0.0044 (10)	−0.0046 (10)	0.0130 (11)
O5	0.0263 (14)	0.096 (3)	0.0377 (16)	0.0102 (17)	0.0005 (12)	−0.0210 (17)
N1	0.0200 (14)	0.0247 (14)	0.0264 (15)	0.0009 (11)	0.0005 (11)	0.0007 (11)
N2	0.0162 (13)	0.0282 (15)	0.0308 (16)	−0.0025 (11)	0.0039 (11)	−0.0015 (12)
Cl1	0.0355 (5)	0.0293 (5)	0.0518 (6)	−0.0015 (4)	−0.0037 (4)	0.0049 (4)

### Geometric parameters (Å, °)

**Table:**

Cu1—O3	1.949 (2)	C6—N2	1.438 (4)
Cu1—O3^i^	2.001 (2)	C7—C8	1.382 (5)
Cu1—N1	2.033 (3)	C7—C10	1.508 (5)
Cu1—O5	2.184 (3)	C8—N1	1.375 (4)
Cu1—Cl1	2.2361 (11)	C8—C9	1.496 (5)
Cu1—Cl1	2.2361 (11)	C9—H9A	0.9600
C1—N1	1.336 (4)	C9—H9B	0.9600
C1—N2	1.365 (4)	C9—H9C	0.9600
C1—C2	1.434 (5)	C10—C11	1.505 (5)
C2—O3	1.320 (4)	C10—H10A	0.9700
C2—C3	1.363 (5)	C10—H10B	0.9700
C3—C4	1.403 (5)	C11—O2	1.419 (5)
C3—H3A	0.9300	C11—H11A	0.9700
C4—C5	1.344 (5)	C11—H11B	0.9700
C4—H4A	0.9300	O2—H2A	0.75 (6)
C5—N2	1.377 (4)	O3—Cu1^i^	2.001 (2)
C5—H5A	0.9300	O5—H5C	0.8200
C6—O1	1.225 (4)	O5—H5D	0.9599
C6—C7	1.407 (5)	Cl1—Cl1	0.000 (3)
			
O3—Cu1—O3^i^	74.24 (11)	N1—C8—C7	122.1 (3)
O3—Cu1—N1	81.74 (10)	N1—C8—C9	116.6 (3)
O3^i^—Cu1—N1	155.95 (11)	C7—C8—C9	121.3 (3)
O3—Cu1—O5	104.82 (13)	C8—C9—H9A	109.5
O3^i^—Cu1—O5	90.24 (11)	C8—C9—H9B	109.5
N1—Cu1—O5	97.00 (11)	H9A—C9—H9B	109.5
O3—Cu1—Cl1	149.96 (9)	C8—C9—H9C	109.5
O3^i^—Cu1—Cl1	95.88 (7)	H9A—C9—H9C	109.5
N1—Cu1—Cl1	104.60 (8)	H9B—C9—H9C	109.5
O5—Cu1—Cl1	103.49 (10)	C11—C10—C7	112.6 (3)
O3—Cu1—Cl1	149.96 (9)	C11—C10—H10A	109.1
O3^i^—Cu1—Cl1	95.88 (7)	C7—C10—H10A	109.1
N1—Cu1—Cl1	104.60 (8)	C11—C10—H10B	109.1
O5—Cu1—Cl1	103.49 (10)	C7—C10—H10B	109.1
Cl1—Cu1—Cl1	0.00 (7)	H10A—C10—H10B	107.8
N1—C1—N2	122.8 (3)	O2—C11—C10	113.2 (3)
N1—C1—C2	118.1 (3)	O2—C11—H11A	108.9
N2—C1—C2	119.1 (3)	C10—C11—H11A	108.9
O3—C2—C3	126.4 (3)	O2—C11—H11B	108.9
O3—C2—C1	114.4 (3)	C10—C11—H11B	108.9
C3—C2—C1	119.2 (3)	H11A—C11—H11B	107.8
C2—C3—C4	119.5 (3)	C11—O2—H2A	111 (5)
C2—C3—H3A	120.3	C2—O3—Cu1	115.4 (2)
C4—C3—H3A	120.3	C2—O3—Cu1^i^	138.3 (2)
C5—C4—C3	121.1 (3)	Cu1—O3—Cu1^i^	105.76 (11)
C5—C4—H4A	119.4	Cu1—O5—H5C	109.5
C3—C4—H4A	119.4	Cu1—O5—H5D	109.3
C4—C5—N2	120.3 (3)	H5C—O5—H5D	109.3
C4—C5—H5A	119.8	C1—N1—C8	118.1 (3)
N2—C5—H5A	119.8	C1—N1—Cu1	110.0 (2)
O1—C6—C7	127.3 (3)	C8—N1—Cu1	131.7 (2)
O1—C6—N2	117.9 (3)	C1—N2—C5	120.7 (3)
C7—C6—N2	114.8 (3)	C1—N2—C6	121.2 (3)
C8—C7—C6	120.9 (3)	C5—N2—C6	118.1 (3)
C8—C7—C10	123.3 (3)	Cl1—Cl1—Cu1	0(10)
C6—C7—C10	115.9 (3)		
			
N1—C1—C2—O3	−0.1 (5)	N2—C1—N1—C8	−0.2 (5)
N2—C1—C2—O3	−179.9 (3)	C2—C1—N1—C8	180.0 (3)
N1—C1—C2—C3	−178.9 (3)	N2—C1—N1—Cu1	−175.7 (3)
N2—C1—C2—C3	1.2 (5)	C2—C1—N1—Cu1	4.4 (4)
O3—C2—C3—C4	179.3 (3)	C7—C8—N1—C1	−1.7 (5)
C1—C2—C3—C4	−2.1 (5)	C9—C8—N1—C1	177.1 (3)
C2—C3—C4—C5	1.3 (6)	C7—C8—N1—Cu1	172.7 (3)
C3—C4—C5—N2	0.4 (6)	C9—C8—N1—Cu1	−8.5 (5)
O1—C6—C7—C8	177.8 (4)	O3—Cu1—N1—C1	−5.3 (2)
N2—C6—C7—C8	−3.3 (5)	O3^i^—Cu1—N1—C1	−2.7 (4)
O1—C6—C7—C10	−0.8 (6)	O5—Cu1—N1—C1	−109.3 (2)
N2—C6—C7—C10	178.0 (3)	Cl1—Cu1—N1—C1	144.7 (2)
C6—C7—C8—N1	3.5 (5)	Cl1—Cu1—N1—C1	144.7 (2)
C10—C7—C8—N1	−177.9 (3)	O3—Cu1—N1—C8	−180.0 (3)
C6—C7—C8—C9	−175.2 (3)	O3^i^—Cu1—N1—C8	−177.5 (3)
C10—C7—C8—C9	3.4 (5)	O5—Cu1—N1—C8	76.0 (3)
C8—C7—C10—C11	−101.0 (4)	Cl1—Cu1—N1—C8	−30.0 (3)
C6—C7—C10—C11	77.6 (4)	Cl1—Cu1—N1—C8	−30.0 (3)
C7—C10—C11—O2	173.1 (3)	N1—C1—N2—C5	−179.5 (3)
C3—C2—O3—Cu1	174.0 (3)	C2—C1—N2—C5	0.4 (5)
C1—C2—O3—Cu1	−4.7 (4)	N1—C1—N2—C6	0.1 (5)
C3—C2—O3—Cu1^i^	3.6 (6)	C2—C1—N2—C6	180.0 (3)
C1—C2—O3—Cu1^i^	−175.1 (2)	C4—C5—N2—C1	−1.2 (5)
O3^i^—Cu1—O3—C2	−173.4 (3)	C4—C5—N2—C6	179.2 (3)
N1—Cu1—O3—C2	5.6 (2)	O1—C6—N2—C1	−179.5 (3)
O5—Cu1—O3—C2	100.7 (2)	C7—C6—N2—C1	1.6 (5)
Cl1—Cu1—O3—C2	−99.4 (3)	O1—C6—N2—C5	0.1 (5)
Cl1—Cu1—O3—C2	−99.4 (3)	C7—C6—N2—C5	−178.8 (3)
O3^i^—Cu1—O3—Cu1^i^	0.0	O3—Cu1—Cl1—Cl1	0.0 (5)
N1—Cu1—O3—Cu1^i^	178.93 (14)	O3^i^—Cu1—Cl1—Cl1	0.0 (5)
O5—Cu1—O3—Cu1^i^	−85.97 (13)	N1—Cu1—Cl1—Cl1	0.0 (5)
Cl1—Cu1—O3—Cu1^i^	74.01 (18)	O5—Cu1—Cl1—Cl1	0.0 (5)
Cl1—Cu1—O3—Cu1^i^	74.01 (18)		

Symmetry codes: (i) −*x*, −*y*+2, −*z*+1.

### Hydrogen-bond geometry (Å, °)

**Table:**

*D*—H···*A*	*D*—H	H···*A*	*D*···*A*	*D*—H···*A*
O5—H5C···O2^ii^	0.82	2.13	2.742 (4)	131.
O5—H5D···O1^iii^	0.96	2.11	2.788 (4)	127.
O2—H2A···Cl1^iv^	0.75 (6)	2.37 (6)	3.078 (4)	158 (6)
C9—H9A···Cl1	0.96	2.49	3.275 (4)	139.
C3—H3A···Cl1^i^	0.93	2.72	3.387 (4)	130.

Symmetry codes: (ii) −*x*+1, −*y*+2, −*z*+2; (iii) *x*−1, *y*, *z*; (iv) *x*+1/2, −*y*+3/2, *z*+1/2;  (i) −*x*, −*y*+2, −*z*+1.
